# Venous thromboembolism prophylaxis protocols at Brazilian hospitals - PROTEV Brazil

**DOI:** 10.1590/1677-5449.190119

**Published:** 2020-06-08

**Authors:** Ana Thereza Cavalcanti Rocha, Thiago Brito Pinheiro, Paulo Roberto Sampaio Peixoto de Souza, Marcos Arêas Marques

**Affiliations:** 1 Universidade Federal da Bahia – UFBA, Faculdade de Medicina da Bahia, Departamento de Saúde da Família, Salvador, BA, Brasil.; 2 Universidade do Estado do Rio de Janeiro – UERJ, Hospital Universitário Pedro Ernesto, Unidade Docente Assistencial de Angiologia, Rio de Janeiro, RJ, Brasil.

**Keywords:** venous thromboembolism, prevention and control, protocols, patient care management, patient safety

## Abstract

**Background:**

In common with other international guidelines, the Agency for Healthcare Research and Quality recommends implementation of venous thromboembolism (VTE) prophylaxis programs in hospitals as a measure for patient safety. The VTE Safety Zone Program (VTESZ) proposes a model for incorporation of systematic VTE risk-assessment into hospital routines, with continuing institutional and multidisciplinary participation.

**Objectives:**

To evaluate implementation of VTE prophylaxis initiatives in Brazilian hospitals that have adhered to the VTESZ Program.

**Methods:**

Questionnaires were e-mailed to VTESZ Program representatives at hospitals visited up to July 2016.

**Results:**

Of the 132 invitations sent, 68 answers were obtained and 50 (73.5%) were complete. 61.5% of participating hospitals had between 100 and 250 beds, and 65.4% had more than 20 intensive care beds; 61.5% reported having hospital accreditation, 86.3% had VTE prophylaxis committees, and 58% had electronic medical records. VTE risk assessments using the Brazilian guidelines or the Padua or Caprini scores were noted on the electronic medical record in 56.9% and were a mandatory step in 45.1% of the cases. VTE risk reassessment was requested prior to discharge in only 25% of hospitals and several issues were cited that negatively affect the VTESZ implementation process.

**Conclusions:**

This study provides an overview of implementation of VTESZ in Brazilian hospitals. Systematic risk assessment is not yet conducted for most patients. Recognition of various issues affecting the process may lead to new strategies for achieving adequate prophylaxis and safety of hospitalized patients.

## INTRODUCTION

Pulmonary thromboembolism (PE) is the third leading cause of cardiovascular mortality worldwide, only coming after acute myocardial infarction (AMI) and stroke.^1^ There are approximately 10 million new cases of venous thromboembolism (VTE) each year globally.^2^ The incidence of VTE may be even higher, because many patients have nonspecific symptoms or mild symptoms of PE or deep venous thrombosis (DVT) and therefore go undiagnosed (“clinically silent VTE”). It is known that the majority of PE episodes (up to 60%) occur during or after hospitalizations,^3^^,^^4^ but the concept that hospital admission itself is a risk factor for VTE, like a nosocomial disease, has not yet been clearly understood by the entire medical community or the population.

In Brazil, although age-adjusted mortality due to PTE has fallen by 31% over the last 21 years, from 3.04/100,000 to 2.09/100,000 inhabitants, there is still considerable variation between Brazil’s five administrative regions, possibly illustrating differences in access to and quality of healthcare in hospitals, or possibly differences in diagnosis or notification.^5^ Nevertheless, once safe and effective methods for prophylaxis exist, VTE became the number one cause of preventable hospital mortality.^6^ However, there are certain obstacles to implementation of VTE prophylaxis in hospitals, one of which is the difficulty of systematizing VTE risk-assessment, both for clinical and for surgical patients. Several studies in Brazilian hospitals have revealed underutilization of prophylaxis in hospitals,^7^^-^9 reaffirming data from the ENDORSE study, according to which, the worldwide mean rate of adequate VTE prophylaxis is just 50% in at-risk medical and surgical patients.^10^

Several simultaneous interventions are recommended to improve adequate VTE prophylaxis in hospitalized patients.^11^^,^^12^ Since publication of the 8th Edition of the American College of Chest Physicians’ (ACCP) clinical practice guidelines for VTE prophylaxis^2^ in 2008, it became clear that a formal program for VTE prophylaxis is not only the responsibility of physicians, but, primarily, the responsibility of the hospital itself. This has been reemphasized in successive recommendations for VTE prophylaxis in specific subgroups of patients and proposals for risk-assessment algorithms or scores based on guideline recommendations^11^^-^14 and by institutions targeting quality.^13^^-^15

The VTE Safety Zone (VTESZ) initiative is a continuing medical education program targeting implementation and optimization of VTE prevention in the hospital settings, to help health professionals to stay alert to VTE risk and transform their hospitals into “VTE-free zones”.

The primary objective of this study, PROTEV Brazil, was to collect data on measures to improve VTE prophylaxis in Brazilian hospitals that have initiated the VTESZ Program, aiming to share strategies to improve the implementation of its recommendations. The study is based on the responses to an electronic questionnaire sent to the professionals responsible for representing the hospitals in which they work.

## METHODS

A quantitative survey was conducted in Brazilian hospitals using an electronic questionnaire comprising 40 multiple-choice questions. The questionnaire was constructed using the on-line platform Survey Monkey® in August 2016 and responses were collected up to July 2017. Potential interviewees were listed by Sanofi® commercial representatives as the contacts responsible for hospitals that had initiated the VTESZ Program. These contacts were invited to participate and complete the questionnaire via e-mail (protev.brasil@gmail.com).

Data about the respondents and their academic qualifications, specialties, and roles at the hospital and on the VTE prophylaxis committee were collected. The second set of questions covered characteristics of the hospitals such as its regional location, type of funding (private, public, philanthropic, or mixed), number of beds, including intensive care beds, type and level of hospital accreditation, proportion of clinical and surgical admissions, and clinical and surgical specialties. The respondents were asked whether their institutions had an electronic patient record system, with the aim of classifying the types of VTE risk-assessment employed, whether assessment was incorporated into the patient medical record, the method of administration, and auditing. Interviewees were asked about the overall level of implementation of the protocol, including continuing care after hospital discharge. Finally, participants described the main barriers affecting implementation of the VTE prophylaxis protocol in their hospitals. Data were tabulated in a spreadsheet using Microsoft Excel® 2010, and responses were organized in the form of percentage frequencies.

## RESULTS

A total of 132 invitations were sent to the nominated contacts at hospitals by e-mail, generating 68 (51.5%) questionnaires returned, 50 (73.5%) of which had been fully answered. Response rates per region varied from 3.8 to 48.1%, with the highest proportion of responding hospitals in the Southeast region (48.1%), followed by the Northeast (25%), South (19.2%), and North and Mid-West regions (both 3.8%).

Thirty of the interviewees 30 (44.1%) were physicians, 10 (33.3%) of whom were intensive care specialists, 6 (20%) were cardiologists, 5 (16.7%) were general practitioners, 4 (13.3%) were general surgeons, and 5 (16.7%) had other specialties. The most frequent profession among those who were not physicians was nursing, with 23 (38.3%) respondents, followed by pharmacy, with 7 (11.7%), and physiotherapy, with 1 (1.7%). The majority of interviewees (46, 76.7%) also had administrative roles in the hospitals they represented.

With regard to type of hospital, 27 (52.9%) were private institutions, 11 (21.6%) were philanthropic, and 7 (13.7%) were public. The majority were considered large hospitals (32, 61.5%), with 100 to 250 beds and more than 20 intensive care beds (34, 65.4%). With regard to the types of patients admitted, 18 (34.6%) hospitals predominantly treated medical patients and 16 hospitals (30.8%) treated equal numbers of medical and surgical patients. The predominant specialties provided at the hospitals were orthopedics and general surgery, both available at 48 (92.3%) hospitals, followed by internal medicine, at 47 (90.4%).

At 29 (58%) hospitals, patient records were electronic, although not necessarily universally accessible at all units or for all purposes, such as medical notes and prescriptions. At 50 (96.1%) institutions, there was an institutional protocol for VTE prophylaxis, 44 (86.3%) respondents mentioned an already-existing and functioning VTE prophylaxis committee, of which the respondent was a member in 42 (82.4%) hospitals. With regard to the VTE risk-assessment tools, algorithms specific for clinical and surgical patients were defined at 47 (92.2%) hospitals, and these were part of the electronic patient record at 29 (56.9%) and were part of a paper-based patient record at 28 (57.1%) hospitals. At 37 (75.5%) hospitals, the VTE risk-assessment algorithm used for clinical patients was the VTESZ Program reference, which corresponds to the recommendations set out in the Brazilian VTE Prophylaxis Guidelines, while the Pádua score was used at 9 (18.4%) hospitals. At 34 (69.4%) hospitals, the VTESZ Program VTE risk-assessment algorithm, which is based on the 9th edition of the ACCP Guidelines,^14^ was used for surgical patients, while 14 (29.6%) hospitals were using the Caprini score. At 23 (45.1%) institutions, completion of the algorithm was obligatory before prescribing. The clinical pharmacy participated in assessment of prophylaxis adequacy at 35 (68.3%) hospitals. Risk-assessment was performed by the nursing team at 33 (64.7%) hospitals and was performed by physicians at 27 (52.9%), or performed by both and validated by physicians at some institutions. At 36 (75%) hospitals, reassessment of VTE risk was not required before hospital discharge, and there was no plan to included the recommendations for VTE prophylaxis post-discharge as a performance indicator.

At 49 (96.1%) hospitals, it was the interviewees’ opinion that the risk-assessment tools chosen for their institutional protocols indicated the correct prophylaxis, although there were criticisms of some of the aspects assessed, such as the estimated length of hospital stay and the definition of reduced mobility as necessary criteria for risk estimation. Participants from 44 (86.3%) hospitals stated that they had had access to performance indicators for the protocol at some point, but did not state which indicators were prioritized for clinical and surgical patients.

With regard to hospital accreditation bodies, the National Accreditation Organization (ONA – Organização Nacional de Acreditação) was the most frequent response (32, 61.5%), followed by QMENTUM or Accreditation Canada (8, 15.4%), and the Joint Commission (7, 13.5%). When asked about the stage of implementation of the managed protocol measures, 11 (21.6%) respondents estimated their hospital to be at an initial stage, 21 (41.2%) at an intermediate stage, and 19 (37.3%) at an advanced stage, although the criteria for these choices were subjective.

Table 1 describes reported issues affecting implementation of the managed protocol, listed in order of occurrence: lack of extended VTE prophylaxis in clinical or oncological patients after hospital discharge (38, 74.5%); lack of VTE prophylaxis protocol for patients discharged from hospital to home care (35, 68.6%); poor physician compliance with completion of risk-assessments (28, 54.9%); software that does not automatically block electronic patient record if the protocol is not filled out (26, 51%); failure to comply with the assessment algorithm even in patients with intermediate/high risk of developing VTE who underwent surgery lasting more than 60 minutes and were discharged from hospital within 48 hours of the procedure (18, 35.3%); failure to follow the prophylaxis protocol during the postoperative period of elective surgery (16, 31.4%); no specific protocol for assessing the risk of pregnant women and during the postpartum period (14, 27.4%); lack of involvement of the nursing team (11, 21.6%); lack of a team member dedicated to managing the protocol (10, 19.6%); failure to incorporate the protocols in all hospital units (10, 19.6%); risk-assessment only obligatory in some units (8, 15.7%); need to adjust the protocol for surgical patients, to avoid overestimating VTE risk (6, 11.8%); and difficulties with implementation of protocol in public hospitals (4, 7.8%).

**Table 1:** Issues affecting implementation of the venous thromboembolism (VTE) prophylaxis protocol (number of responses = 51).

**Responses**	**n (%)**
Prophylaxis maintenance for clinical or oncological patients after hospital discharge	38 (74.5%)
Lack of a VTE prophylaxis protocol for patients discharged from hospital/to home care	35 (68.6%)
Poor compliance with completion of risk assessments by physicians	28 (54.9%)
Lack of an automatic software lock on electronic patient record to oblige completion of the protocol	26 (51%)
Failure to adhere to surgical protocol in patients with intermediate/high risk and length of hospital stay < 48 h (duration of surgery > 60 min)	18 (35.3%)
Failure to follow the prophylaxis protocol during the postoperative period of elective surgery	16 (31.4%)
No specific VTE protocol for obstetrics	14 (27.5%)
Lack of involvement of the nursing team	11 (21.6%)
Protocol only exists in paper-based format	11 (21.6%)
Lack of a team member dedicated to managing the protocol	10 (19.6%)
Failure to cascade the protocols to all hospital units	10 (19.6%)
Risk assessment only obligatory in some units	8 (15.7%)
Need to adjust the protocol for surgical patients, to avoid overestimating VTE risk	6 (11.8%)
Issues with initiating the protocol in public hospitals	4 (7.8%)

## DISCUSSION

Several guidelines and institutions recommend implementing a formal program for VTE prophylaxis in hospitals to ensure patient safety.^14^^-^16 However, to do so successfully demands institutional and multidisciplinary participation and continuing education. The VTESZ Program is a global continuing medical education program focused on implementation and optimization of VTE prevention in hospital settings that is aimed at physicians and other health professionals. It was initiated in 2007 in Brazil by the Sanofi® laboratory, with scientific support from the Brazilian Thoracic Association (Sociedade Brasileira de Pneumologia e Tisiologia). The program’s primary objective is to raise health professionals’ awareness about VTE risk and help them to transform their hospitals into “VTE-free zones” using strategies to solve issues of underutilization and inadequacy of VTE prophylaxis, implementing the recommendations of evidence-based guidelines. Box 1 lists the key recommendations for prevention of VTE in hospitals according to the 8th VTE Prophylaxis Guidelines from the ACCP,^2^ which are the foundation of the program’s strategies.

**Box 1:** Key recommendations from the American College of Chest Physicians (ACCP) on prevention of venous thromboembolism (VTE) in hospitals.

1) Every hospital should develop a formal strategy that addresses the prevention of VTE (grade 1A) and, preferably, have a written hospital policy or protocol for implementation throughout the entire institution (grade 1C);
2) Passive distribution of educational material or educational lectures are not recommended in isolation as strategies for increasing compliance with VTE prophylaxis (grade 1B);
3) Recommended strategies for increasing compliance with VTE prophylaxis should include:
a) computerized systems to support risk assessment and prescription (grade 1A);
b) protocols with standardized prescriptions (grade 1B);
c) proactive committees that conduct periodic audits of VTE prophylaxis use and present results to the institution’s clinical care teams (grade 1C).

A systematic review of interventions to improve VTE prophylaxis in hospitals showed that programs with multiple strategies are most effective.^17^ A cross-sectional study compared proportions of patients at VTE risk before and after implementation of VTE prophylaxis programs in four hospitals in Salvador^18^ and analyzed changes in rates of adequate prophylaxis, assessing 219 clinical patients before and 292 after implementation of a program comprising continuing education and passive distribution of printed algorithms for VTE risk-assessment. The study showed that there was an increase in the percentage of patients considered candidates for prophylaxis from the first to the second data collections, from 75% to 82% (p = 0.06), and also an increase in the proportion of patients without any contraindications for heparin use, from 44% to 55% (p = 0.02). After the program, mechanical prophylaxis was being used more frequently, 0.9% vs. 4.5% (p = 0.03), and there was a significant increase in use of the correct doses of heparins, 53% vs. 75% (p < 0.001). However, although rates of adequate VTE prophylaxis had improved, it remained underutilized in the hospitals evaluated, showing that the process of implementation needs to be incorporated into hospital routines, preferably in a manner that systematically alerts professionals. Curtarelli et al.^11^ conducted a cross-sectional study at a university hospital, finding that 57.9% of 456 medical and surgical patients analyzed did not receive adequate VTE prophylaxis and that this was more frequent among surgical patients (62.5%) and was very often in the form of not prescribing pharmacological prophylaxis, particularly to those with moderate risk. In that study, there was no availability of mechanical prophylaxis methods such as graduated compression elastic stockings or intermittent pneumatic compression, which are feasible options, particularly for subsets of surgical patients at moderate risk. There were more errors in choice and dose of medications among the medical patients assessed in the study and a tendency was identified to use direct factor IIa or Xa anticoagulants in subsets for which these grugs have not yet been formally indicated in guidelines based on scientific evidence from suitably-sized studies. Nevertheless, although overuse of prophylaxis occurred in 4.8% of patients with anticoagulant prescriptions for whom this was not indicated, this problem is still less common than underutilization, but can create additional costs and risks.

The VTESZ Program^12^ provides several tools that can and should be used in conjunction. The first step is to have someone at the hospital to lead the program that advocates for the VTE prophylaxis cause and controls the dynamics of the process. It is very important to obtain the support of top hospital management to ensure buy-in with the measures that must be implemented into medical units’ routines. A cross-sectional study, conducted as an audit, can provide information on the baseline situation at the hospital with relation to use of VTE prophylaxis, highlighting specialties or wards that merit special attention. Another very important point is to create a committee, preferably multidisciplinary, to encourage the hospital’s staff to practice VTE prophylaxis. Several members of the clinical staff should be involved, in addition to representatives from medical specialties and surgical specialties, including pharmacy, nursing, physiotherapy, and the quality care assurance team, etc. It is also important to promote systematic completion of VTE risk-assessment instruments, such as electronic or paper-based algorithms, or standardized prescriptions in certain high-risk services, such as orthopedics and intensive care. These algorithms should be evidence-based and agreed upon by the local VTE prophylaxis committee.

In the present study, it was found that the majority of the hospitals that were attempting to implement VTE prophylaxis programs such as TEVSZ are large, already involved in accreditation to improve quality, and offer care in multiple specialties, serving a majority of patients with high VTE risk. Several professionals are involved in the VTE prophylaxis committees and they are attempting to integrate medical and surgical risk-assessment algorithms into their patient records, in a manner that is customized at each hospital, but not always systematically across all units, or with electronic patient records that automatically alert prescribers. Several issues that were encountered during the processes of implementation and maintenance of the measures were described. A minority of the hospitals analyzed are public or philanthropic and they faced additional obstacles in the form of lack of electronic patient record systems that could provide automatic alerts and block prescriptions if the VTE risk-assessment is not performed, in addition to a lack of personnel assigned to manage the protocol. At the hospitals at more advanced stages of implementation, more specific and detailed issues impacting on adequate prophylaxis were described; related to subsets of medical patients at moderate risk, to continuation of prophylaxis in high risk patients after hospital discharge (for example, cancer/oncology patients surgical), and to lack of consensus or protocols on the best forms of assessment and VTE prevention for other groups of patients (for example, chronically ill patients and during pregnancy and postpartum).

This study has certain limitations and biases, such as the fact that the responses were provided by representatives who had mediated the initial program activities and were nominated by consultants from Sanofi®. The response rate of hospitals contacted was 51.5%. In view of the high turnover of hospital staff, it is likely that several of these professionals were no longer in the same roles or no longer responsible for the program being implemented. Only hospitals that had already expressed interest in launching a formal prophylaxis program were included and it is probable that the situation at other hospitals in Brazil is different and less advanced in terms of these quality measures, of systematic VTE risk-assessment, and of adequacy of VTE prophylaxis. Moreover, the majority of the hospitals surveyed were large (with more than 100 beds), which is representative of a minority (18.7%) of the 7,514 hospitals in the Brazilian hospital network.^19^ It is very likely that small hospitals, with 1 to 49 beds (4,576 hospitals, 60.9% of the total), and medium-sized hospitals, with 50 to 100 beds (1,535 hospitals, 20.4% of the total), have even less capacity to implement and manage a VTE prophylaxis program. This illustrates a problem with the coverage of quality and VTE prophylaxis programs, since small hospitals constitute the hospitals available in 2,785 municipal districts in Brazil and provide 69% of their beds to the Brazilian state healthcare system, the SUS.^19^

Another limitation of this study is the subjectivity with which levels of implementation of the VTESZ Program were defined by the interviewees. It is known that well-implemented and organized processes are dependent on interaction and integration between multidisciplinary teams and on institutional support to standardize procedures and set up safety policies, which are then measured in terms of performance results. According to the Agency for Healthcare Research and Quality,^6^ there is a hierarchy of five levels of reliability of implementation and complexity of processes for VTE prophylaxis. Level 1, or baseline, encompasses hospitals still in their “natural state” where VTE prophylaxis adequacy rates are around 40%. Level 2, the initial level, suggests that a protocol exists, but is not being incorporated into daily practice from admission onwards or into stages of patient transfer between inpatient units, with rates of around 50%. Level 3, the intermediate level, suggests that the protocol is well integrated into the stages of care and prophylaxis adequacy rates are in the range of 60 to 85%. Level 4, the advanced level, denotes that the protocol has been adapted into other strategies for quality improvement, raising prophylaxis rates to 90%. Finally, level 5, the ideal state, involves a protocol that identifies omissions and corrects prophylaxis in real time, achieving VTE prophylaxis rates in excess of 95%.

## CONCLUSIONS

When listing issues with implementation of VTE prophylaxis measures faced by hospitals that have initiated the VTESZ Program, we observed certain barriers that need to be dealt with. There are still knowledge gaps in the guidelines in terms of approach and managment for some subsets of patients, raising the need for guidance regarding risk-assessment and use of prophylaxis for patients at potential risk, but for whom there is underutilization of prophylaxis, such as pregnant and postpartum women and chronically ill patients with reduced mobility. It was also perceived that there are ongoing tasks, at each institution seeking its own solutions to overcome these issues. The guidance from institutions such as the Brazilian Institute for Patient Safety (IBSP - Instituto Brasileiro para Segurança do Paciente)^16^ could improve the search for solutions, targeting real-time monitoring of VTE prevention and interventions to reduce thromboembolic events and readmissions for VTE.
