# Supplementary material for: Venous thromboembolism prophylaxis protocols at Brazilian hospitals - PROTEV Brazil
Source: J Vasc Bras. 2020 Jun 8;19:e20190119. doi: 10.1590/1677-5449.190119 (PMC8202209; doi:10.1590/1677-5449.190119)
Supplement: Translation

# Translation: Protocolos de profilaxia de tromboembolismo venoso (TEV) em hospitais brasileiros - PROTEV Brasil

**Authors:** Ana Thereza Cavalcanti Rocha, Thiago Brito Pinheiro, Paulo Roberto Sampaio Peixoto de Souza, Marcos Arêas Marques

**Affiliations:** 1 Universidade Federal da Bahia – UFBA, Faculdade de Medicina da Bahia, Departamento de Saúde da Família, Salvador, BA, Brasil.; 2 Universidade do Estado do Rio de Janeiro – UERJ, Hospital Universitário Pedro Ernesto, Unidade Docente Assistencial de Angiologia, Rio de Janeiro, RJ, Brasil.

**Keywords:** tromboembolismo venoso, prevenção e controle, protocolos, administração dos cuidados ao paciente, segurança do paciente

## Abstract

**Contexto:**

Implementar um programa para profilaxia de tromboembolismo venoso (TEV) em hospitais é uma recomendação de diretrizes internacionais e da *Agency for Healthcare Research and Quality* para segurança dos pacientes. O Programa TEV *Safety Zone* (TEVSZ) é um modelo que sugere avaliação sistemática do risco de TEV incorporada às rotinas do hospital com participação institucional e multidisciplinar continuada.

**Objetivos:**

Levantar dados de implementação de iniciativas para profilaxia em hospitais brasileiros que iniciaram o Programa TEVSZ.

**Métodos:**

Envio de questionário por correio eletrônico aos responsáveis pelos programas TEVSZ em hospitais visitados até julho de 2016.

**Resultados:**

Dos 132 convites enviados, foram obtidas 68 respostas, sendo 50 (73,5%) completas. Em 61,5% dos hospitais participantes havia entre 100 e 250 leitos, e 65,4% tinham mais de 20 leitos de terapia intensiva; 61,5% referiam ter acreditação hospitalar, 86,3% tinham comissão de profilaxia de TEV e 58% tinham prontuários eletrônicos. As avaliações de risco de TEV pela diretriz brasileira, escores de Pádua ou Caprini eram feitas no prontuário eletrônico em 56,9% e como passo obrigatório em 45,1% dos casos. Em apenas 25% dos hospitais, a reavaliação do risco de TEV era solicitada antes da alta, e foram citadas várias barreiras no processo de implementação do TEVSZ.

**Conclusões:**

O estudo mostra um panorama da implementação do TEVSZ em hospitais brasileiros. As avaliações sistemáticas de risco ainda não ocorrem na maioria dos pacientes. O reconhecimento de diversas barreiras no processo pode levar a novas estratégias para a adequação da profilaxia e segurança dos pacientes hospitalizados.

## INTRODUÇÃO

O tromboembolismo pulmonar (TEP) é a terceira causa de mortalidade cardiovascular no mundo, atrás apenas de infarto agudo do miocárdio (IAM) e acidente vascular cerebral (AVC)^1^. Há aproximadamente 10 milhões de novos casos anuais de tromboembolismo venoso (TEV) no mundo^2^. A incidência de TEV pode ser ainda maior porque muitos pacientes apresentam sintomas inespecíficos ou sintomas leves de TEP ou trombose venosa profunda (TVP), não sendo diagnosticados (“TEV clinicamente silenciosa”). Sabe-se ainda que a maioria dos episódios de TEP (até 60%) ocorre durante ou após a hospitalização^3^^,^^4^, mas o conceito de que a internação hospitalar representa um fator de risco para o TEV, como uma doença nosocomial, ainda não é claramente entendido por toda a comunidade médica ou pela população.

No Brasil, apesar da ter havido redução de 31% na mortalidade ajustada por idade devida ao TEP nos últimos 21 anos, de 3,04/100.000 para 2,09/100.000 habitantes, ainda há variação importante entre as cinco regiões brasileiras, denotando possivelmente diferença de acesso e qualidade da saúde em hospitais ou talvez de diagnóstico ou notificação^5^. Por outro lado, existem métodos eficazes e seguros de profilaxia, tornando o TEV a causa número um de morte hospitalar prevenível^6^. Entretanto, há algumas barreiras para a implementação da profilaxia do TEV em hospitais, sendo uma delas a dificuldade em sistematizar a avaliação do risco de TEV, tanto em pacientes clínicos quanto em cirúrgicos. Vários estudos em hospitais brasileiros mostram subutilização da profilaxia em hospitais^7^^-^9, reiterando dados do estudo ENDORSE, segundo o qual, a nível mundial, a média da adequação de profilaxia de TEV é apenas de 50% em pacientes clínicos e cirúrgicos com alto risco^10^.

Várias intervenções simultâneas para melhoria da adequação da profilaxia do TEV em pacientes hospitalizados são recomendadas^11^^,^^12^. Desde a publicação da 8^a^ Edição da Diretriz para a Prática Clínica do *American College of Chest Physicians* (ACCP) sobre a Profilaxia de TEV^2^, em 2008, ficou claro que ter um programa formal de profilaxia de TEV é responsabilidade não só do médico, mas principalmente do hospital. Isso vem sendo reiterado em recomendações específicas para profilaxia em subgrupos de pacientes e sugestões de algoritmos ou escores de avaliação por diretrizes^11^^-^14 e por instituições que visam a qualidade^13^^-^15.

O TEV *Safety Zone* (TEVSZ) é um programa global de educação médica continuada para implementação e otimização da prevenção do TEV no ambiente hospitalar, para auxiliar os profissionais de saúde a ficarem alertas sobre o risco de TEV e transformarem seus hospitais em “zonas livres de TEV”.

O objetivo principal deste estudo, **PROTEV Brasil**, foi levantar dados sobre os cuidados com a profilaxia de TEV em hospitais brasileiros que iniciaram o Programa TEVSZ, visando a compartilhar estratégias para melhorias na implementação das recomendações, baseando-se nas respostas de questionário eletrônico enviado aos profissionais responsáveis por representarem o hospital onde trabalham.

## MÉTODOS

Foi realizada uma pesquisa quantitativa por questionário eletrônico com 40 questões de múltipla escolha em hospitais do território nacional. O questionário foi criado a partir da plataforma *on-line* Survey Monkey® em agosto de 2016, sendo as respostas coletadas até julho de 2017. Os entrevistados foram apontados por representantes comerciais da Sanofi® como contatos responsáveis nos hospitais que iniciaram o Programa TEVSZ, sendo convidados por meio de correio eletrônico (protev.brasil@gmail.com) para participação e preenchimento do questionário.

Foram coletados dados do participante sobre sua área de formação, especialidade, atuação na unidade hospitalar e na comissão de profilaxia de TEV. As demais questões caracterizaram os hospitais quanto a localização regional, tipo de gestão da unidade (privada, pública, filantrópica ou mista), número de leitos de internação, incluindo de terapia intensiva, tipo e nível de acreditação hospitalar, proporção de internações clínicas ou cirúrgicas, especialidades clínicas e cirúrgicas existentes. Foram questionados sobre a existência de prontuário eletrônico, visando a caracterizar os tipos de avaliações de risco de TEV utilizadas, incorporação no prontuário, modo de aplicação e auditoria. Os entrevistados foram indagados sobre o nível de implementação do protocolo como um todo, incluindo o cuidado continuado na alta hospitalar. Finalmente, os participantes relataram as principais barreiras que dificultavam a implementação do protocolo de profilaxia de TEV no hospital. Os dados foram tabulados em planilha do *software* Microsoft Excel® 2010, no qual foram organizadas as respostas na forma de frequência percentual.

## RESULTADOS

Entre os 132 convites enviados por correio eletrônico aos contatos hospitalares, foram obtidas 68 (51,5%) respostas ao questionário, sendo 50 (73,5%) completas. As taxas de respostas oscilaram entre 3,8 e 48,1% entre as regiões do país, sendo a Região Sudeste a que teve o maior número de hospitais participantes (48,1%), seguida das regiões Nordeste (25%), Sul (19,2%) e, por fim, Norte e Centro-Oeste (3,8% em ambas).

Entre os entrevistados, 30 (44,1%) eram médicos, sendo que, destes, 10 (33,3%) eram intensivistas, 6 (20%) cardiologistas, 5 (16,7%) clínicos em geral, 4 (13,3%) cirurgiões gerais e 5 (16,7%) tinham outras especialidades. Entre os não médicos, a área de formação mais frequente foi enfermagem, com 23 (38,3%) indivíduos, seguida de farmácia, com 7 (11,7%), e fisioterapia, com 1 (1,7%). A maioria dos entrevistados, 46 (76,7%), exercia também funções administrativas no hospital representado.

Quanto à caracterização dos hospitais, 27 (52,9%) eram instituições privadas, 11 (21,6%) eram filantrópicas e 7 (13,7%) eram públicas. A maior parte era considerada hospital de grande porte (32, 61,5%), com 100 a 250 leitos de internação e mais do que 20 leitos de terapia intensiva (34, 65,4%). No que tange ao perfil das internações, predominavam pacientes clínicos em 18 hospitais (34,6%) e proporcionais entre pacientes clínicos e cirúrgicos em 16 hospitais (30,8%). Entre as especialidades contempladas em cada hospital, ortopedia e cirurgia geral predominaram, ambas presentes em 48 (92,3%) hospitais, seguidas de clínica geral em 47 (90,4%).

Em 29 (58%) hospitais, os prontuários médicos eram eletrônicos, embora não universalmente disponíveis em todas as unidades ou para todos os propósitos como evoluções e prescrições. Em 50 (96,1%) instituições, existia um protocolo institucional de profilaxia de TEV, sendo que, em 44 (86,3%), foi citada a existência de comissão de profilaxia de TEV já montada e que incluía o participante em 42 (82,4%) hospitais. Quanto às ferramentas de avaliação do risco de TEV, havia algoritmos definidos para pacientes clínicos e cirúrgicos em 47 (92,2%) hospitais, fazendo parte do prontuário eletrônico em 29 (56,9%) e em prontuários impressos em 28 (57,1%) hospitais. O algoritmo de avaliação de risco de TEV utilizado em 37 (75,5%) hospitais para pacientes clínicos foi a referência do Programa TEVSZ, que corresponde às recomendações da Diretriz Brasileira de Profilaxia de TEV, seguido do escore de Pádua em 9 (18,4%). Para 34 (69,4%) hospitais, foi aplicado o algoritmo de avaliação de risco de TEV em pacientes cirúrgicos do Programa TEVSZ, baseado na 9^a^ edição da Diretriz do ACCP^14^ e, para 14 (29,6%), o escore de Caprini. Em 23 (45,1%) instituições, o preenchimento do algoritmo era obrigatório antes da realização das prescrições. A farmácia clínica participava da avaliação da adequação da profilaxia em 35 (68,3%) hospitais. A avaliação de risco era realizada pela enfermagem em 33 (64,7%) unidades e por médicos em 27 (52,9%), havendo superposição de avaliação ou validação pelo médico em algumas instituições. Em 36 (75%) hospitais, a reavaliação do risco de TEV não era solicitada antes da alta hospitalar do paciente, não havendo também estabelecimento de plano que incluísse recomendações sobre profilaxia do TEV pós-alta na maior parte destes como um indicador de desempenho.

A percepção dos entrevistados é de que as ferramentas de avaliação de risco escolhidas para o protocolo indicaram a profilaxia correta em 49 (96,1%) hospitais, havendo críticas a alguns aspectos avaliados, como tempo estimado de internação e da definição quanto à redução da mobilidade como critério necessário para estimativa de risco. Os participantes relataram já terem tido acesso em algum momento aos indicadores de desempenho do protocolo em 44 (86,3%) hospitais, mas não foram especificados quais os indicadores priorizados para pacientes clínicos e cirúrgicos.

Quanto ao tipo de acreditação hospitalar, a Organização Nacional de Acreditação (ONA) foi a mais citada (32, 61,5%), seguida da QMENTUM ou Canadense (8, 15,4%) e da *Joint Commission* (7, 13,5%). Quanto ao estágio de implementação das medidas do protocolo gerenciado, 11 (21,6%) estimavam que o hospital estava em estágio inicial, 21 (41,2%), em estágio intermediário e 19 (37,3%), em fase avançada, embora os critérios para isso tenham sido subjetivos.

As dificuldades apontadas para a implementação do protocolo gerenciado são mostradas na Tabela 1, sendo elas descritas em ordem de ocorrência: falta de manutenção da profilaxia em pacientes clínicos ou oncológicos após a alta hospitalar (38, 74,5%); falta de protocolo de profilaxia de TEV para pacientes de alta hospitalar/assistência domiciliar (35, 68,6%); má adesão dos médicos ao preenchimento das avaliações de risco (28, 54,9%); não implementação de bloqueio de prontuário eletrônico por *software* na ausência de preenchimento do protocolo (26, 51%); falta de adesão ao algoritmo de avaliação mesmo em pacientes de risco intermediário/alto para o desenvolvimento de TEV que foram submetidos a cirurgias com duração superior a 60 minutos e que receberam alta hospitalar em até 48 horas após o procedimento (18, 35,3%); não cumprimento do protocolo durante profilaxia no pós-operatório de cirurgias eletivas (16, 31,4%); inexistência de protocolo específico para avaliação de risco para gestantes e puérperas (14, 27,4%); falta de envolvimento da equipe de enfermagem (11, 21,6%); ausência de membro da equipe dedicado ao gerenciamento do protocolo (10, 19,6%); falta de disseminação dos protocolos entre todas as unidades hospitalares (10, 19,6%); obrigatoriedade de avaliação de risco em apenas algumas unidades (8, 15,7%); necessidade de ajustes do protocolo para pacientes cirúrgicos, a fim de que seja evitada supervalorização do risco de TEV (6, 11,8%); dificuldades de implementação do protocolo em hospitais públicos (4, 7,8%).

**Tabela 1:** Barreiras encontradas para a implementação do protocolo de profilaxia de tromboembolismo venoso (TEV) (número de respostas = 51).

**Respostas**	**n (%)**
Manter profilaxia na alta hospitalar para pacientes clínicos/oncológicos	38 (74,5%)
Falta de protocolo de profilaxia de TEV para pacientes de alta hospitalar/assistência domiciliar	35 (68,6%)
Falta de adesão ao preenchimento do protocolo pelos médicos	28 (54,9%)
Falta de instalação do bloqueio de prontuário eletrônico para tornar o preenchimento do protocolo obrigatório	26 (51%)
Falta de adesão ao protocolo cirúrgico em pacientes com risco intermediário/alto e tempo de permanência hospitalar < 48 h (duração de cirurgias > 60 min)	18 (35,3%)
Falta de preenchimento do protocolo de profilaxia no pós-operatório de cirurgias eletivas	16 (31,4%)
Falta de protocolo de TEV específico para obstetrícia	14 (27,5%)
Falta de envolvimento da equipe de enfermagem	11 (21,6%)
Protocolo existe apenas no papel	11 (21,6%)
Falta de membro da equipe dedicado ao protocolo	10 (19,6%)
Falta de disseminação dos protocolos entre as unidades hospitalares	10 (19,6%)
Avaliação obrigatória de risco apenas em algumas unidades	8 (15,7%)
Protocolo para pacientes cirúrgicos precisa de ajustes para evitar superclassificação de risco de TEV	6 (11,8%)
Iniciar protocolo em hospital público	4 (7,8%)

## DISCUSSÃO

Implementar um programa formal para a profilaxia de TEV em hospitais é uma recomendação de inúmeras diretrizes e instituições para garantir a segurança dos pacientes^14^^-^16. Exige, porém, participação institucional e multidisciplinar e educação continuada para obter êxito. O Programa TEVSZ é um programa global de educação médica continuada para implementação e otimização da prevenção do TEV no ambiente hospitalar, dirigido a médicos e a outros profissionais de saúde. Ele foi iniciado no Brasil em 2007 pelo laboratório Sanofi® com embasamento científico da Sociedade Brasileira de Pneumologia e Tisiologia. O objetivo principal do programa é auxiliar os profissionais de saúde a ficarem alertas sobre o risco de TEV e transformarem seus hospitais em “zonas livres de TEV” por meio de estratégias para solucionar as questões da subutilização e inadequação da profilaxia de TEV, implementando recomendações de diretrizes baseadas em evidências clínicas. No Quadro 1, são listadas as recomendações-chave sobre a prevenção do TEV em hospitais pela 8^a^ Diretriz de Profilaxia do ACCP^2^, as quais são a base das estratégias deste programa.

**Quadro 1:** Recomendações-chave do *American College of Chest Physicians* (ACCP) sobre a prevenção do tromboembolismo venoso (TEV) em hospitais.

1) Todo hospital deve desenvolver uma estratégia formal para abordar a prevenção do TEV (grau 1A) e, de preferência, deve ter uma política hospitalar ou protocolo, por escrito, para implementação em toda a instituição (grau 1C);
2) A distribuição passiva de materiais educacionais ou palestras educativas não são recomendadas, como estratégias isoladas, para aumentar a aderência à profilaxia do TEV (grau 1B);
3) As estratégias recomendadas para aumentar a aderência ao uso de profilaxia do TEV devem incluir:
a) sistemas computadorizados de suporte à avaliação de risco e à prescrição (grau 1A);
b) protocolos com prescrições padronizadas (grau 1B);
c) comissões proativas que realizem auditorias periódicas sobre o uso de profilaxia do TEV e apresentem resultados para o corpo clínico da instituição (grau 1C).

Uma revisão sistemática de intervenções para melhorar a profilaxia de TEV em hospitais mostrou que programas com estratégias múltiplas são mais efetivos^17^. Em estudo de corte transversal, antes e depois da implementação de programas de profilaxia de TEV em quatro hospitais de Salvador^18^, foram comparadas as proporções de pacientes em risco de TEV e as mudanças na adequação da profilaxia, sendo avaliados 219 pacientes clínicos antes e 292 depois do programa que contava com aulas de educação continuada e distribuição passiva de algoritmos impressos de avaliação do risco de TEV. Mostrou-se que, entre os dois estudos, houve um aumento do percentual de pacientes considerados candidatos para profilaxia 75% *versus* 82% (p = 0,06) e daqueles sem qualquer contraindicação para uso de heparinas, 44% *versus* 55% (p = 0,02). Após o programa, utilizou-se mais profilaxia mecânica, 0,9% *versus* 4,5% (p = 0,03) e houve um aumento significativo na utilização das doses corretas das heparinas, 53% *versus* 75% (p < 0,001). Apesar de haver melhor adequação da profilaxia de TEV, ela permaneceu subutilizada nos hospitais avaliados, mostrando que o processo de implementação necessita ser incorporado às rotinas hospitalares, preferencialmente de modo a alertar sistematicamente aos profissionais. No estudo de corte transversal de Curtarelli et al.^11^ em hospital universitário, entre os 456 pacientes clínicos e cirúrgicos analisados, houve 57,9% de inadequação da profilaxia de TEV, sendo mais pronunciada entre os pacientes cirúrgicos (62,5%) e devida muitas vezes à não prescrição de profilaxia química, particularmente entre aqueles com risco moderado. Neste estudo, não houve disponibilidade de métodos mecânicos de profilaxia como meias elásticas de compressão gradual ou compressão pneumática intermitente, que são opções viáveis particularmente para subgrupos de pacientes cirúrgicos de risco moderado. Quanto aos pacientes clínicos avaliados neste estudo, houve mais erros na escolha da medicação e na dose, o que alerta sobre a tendência de utilização de anticoagulantes diretos do fator IIa ou Xa em subgrupos para os quais ainda não há indicação formal de diretrizes, com embasamento científico de estudos de porte adequado. Além disso, embora a superutilização da profilaxia tenho ocorrido em 4,8% dos pacientes com prescrição de anticoagulante, mas sem indicação, este ainda é um problema menos frequente que a subutilização e que pode gerar custos e riscos adicionais.

O Programa TEVSZ^12^ disponibiliza várias ferramentas que podem e devem ser utilizadas em conjunto. O primeiro passo é ter alguém no hospital como líder do programa que abrace a causa da profilaxia do TEV e conduza a dinâmica desse processo. É muito importante ter apoio da diretoria do hospital para embasar as medidas que devem ser implementadas como rotinas nos serviços. A realização de um estudo de corte transversal, como uma auditoria, pode trazer informações sobre a realidade basal da utilização de profilaxia do TEV no hospital em questão, servindo para destacar especialidades ou enfermarias que merecem maior atenção. Outro ponto muito importante é criar uma comissão, de preferência multidisciplinar, para incentivar os profissionais a praticar a profilaxia do TEV. Vários membros do corpo clínico, além de um representante da clínica e outro das especialidades cirúrgicas, devem ser envolvidos, como farmacêuticos, enfermeiros, fisioterapeutas, membros da qualidade de atendimento, etc. Além disso, é importante promover o preenchimento sistemático das ferramentas de avaliação de risco para TEV, como os algoritmos eletrônicos ou em papel, ou prescrições padronizadas em alguns serviços de alto risco, como a ortopedia e a terapia intensiva. Esses algoritmos devem estar baseados em evidências e orientados pela comissão local de profilaxia de TEV.

No presente estudo, foi demonstrado que os hospitais que buscaram implementar programas de profilaxia de TEV como o TEVSZ são, na sua maioria, grandes, já envolvidos em acreditação para melhoria da qualidade e oferecem atendimentos de múltiplas especialidades, incluindo a maioria dos pacientes com alto risco de TEV. Vários profissionais estão envolvidos nas comissões de profilaxia de TEV e procurou-se incluir os algoritmos de avaliação de risco clínico e cirúrgico nos prontuários, de modo customizado para cada hospital, porém, nem sempre de modo sistemático em todas as unidades, ou em prontuários eletrônicos, para alertar automaticamente os prescritores. Ao longo do processo de implementação e manutenção das medidas, foram citadas diversas barreiras. A minoria dos hospitais avaliados são públicos ou filantrópicos, sendo que nestes destacaram-se como barreiras adicionais a falta de prontuário eletrônico que possibilitasse alertas automáticos e bloqueios na prescrição caso a avaliação do risco de TEV não fosse realizada e a falta de pessoal dedicado para o gerenciamento do protocolo. Entre os hospitais em fases mais avançadas de implementação, foram citadas barreiras mais específicas e pormenorizadas para a adequação de profilaxia em subgrupos de pacientes clínicos de risco moderado, a continuação da profilaxia em pacientes de alto risco após a alta hospitalar (por exemplo, pacientes oncológicos cirúrgicos) e outros grupos de pacientes para os quais não há consenso ou protocolo sobre a melhor forma de avaliação e prevenção do TEV (por exemplo, pacientes cronicamente enfermos e para gestantes e puérperas).

O estudo tem algumas limitações e vieses, como o fato de que as respostas vieram de representantes que mediaram as atividades iniciais do programa, sendo apontados pelos consultores do laboratório Sanofi®. A taxa de resposta dos hospitais contatados foi de 51,5%. Devido à rotatividade inerente às equipes profissionais em hospitais, é provável que vários destes profissionais não estivessem nas mesmas posições ou responsáveis por responder pelo programa em implementação. Apenas os hospitais que já tinham o intuito de iniciar um programa formal de profilaxia foram incluídos e provavelmente a situação de outros hospitais do país seja diferente e menos avançada quanto a estas medidas de qualidade, à avaliação sistemática de risco de TEV e ao uso adequado de profilaxia. De fato, a maioria dos hospitais contemplados eram grandes (com mais de 100 leitos), o que representa apenas a minoria, ou seja, 18,7% dos 7.514 hospitais da Rede Hospitalar Brasileira^19^. Novamente, ressalta-se a probabilidade de que hospitais pequenos, com 1 a 49 leitos (4.576 hospitais, 60,9% do total) e médios, com 50 a 100 leitos (1.535, 20,4% do total), tenham menor capacidade ainda de iniciar e gerenciar um programa de profilaxia de TEV. Isto ilustra um problema de alcance das medidas de programas de qualidade e profilaxia de TEV, já que os hospitais pequenos representam as unidades hospitalares disponíveis em 2.785 municípios brasileiros e oferecem 69% dos seus leitos para o SUS^19^.

Outra limitação do estudo foi a subjetividade com a qual foram definidos os níveis de implementação do Programa TEVSZ pelos entrevistados. É sabido que processos bem implantados e estruturados dependem de articulação e integração entre equipes multidisciplinares e apoio institucional para padronização de procedimentos e implantação de políticas de segurança, sendo medidos através de resultados de desempenho. De acordo com a *Agency for Healthcare Research and Quality* 6, existe uma hierarquia de cinco níveis de confiabilidade da implementação e complexidade dos processos para a profilaxia de TEV. O nível 1, ou basal, reflete hospitais ainda em seu “estado natural” quanto à adequação das taxas de profilaxia de TEV, em cerca de 40%. O nível 2, ou inicial, sugere que o protocolo existe, mas não está incorporado à prática diária desde a admissão ou as etapas de transferência do paciente entre as unidades de internação, com taxas de cerca de 50%. O nível 3, ou intermediário, sugere que o protocolo está bem integrado às etapas dos cuidados e as taxas de adequação de profilaxia oscilam entre 60 e 85%. O nível 4, ou avançado, denota que o protocolo está ajustado com as outras estratégias de melhoria da qualidade, levando a taxas de profilaxia de 90%. Finalmente, o nível 5, ou ideal, apresenta protocolo que identifica a omissão e corrige a profilaxia em tempo real, visando a taxas de profilaxia de TEV maiores que 95%.

## CONCLUSÃO

Ao listarmos as barreiras quanto a implementação das medidas para profilaxia de TEV em hospitais que iniciaram o Programa TEVSZ, percebemos algumas dificuldades existentes e que precisam ser abordadas. Há ainda lacunas no conhecimento e abordagem em diretrizes, sendo necessário o estabelecimento de guias de conduta quanto à avaliação de risco e ao uso de profilaxia em subgrupos específicos, com risco potencial, mas com subutilização de profilaxia, como gestantes, puérperas e pacientes cronicamente enfermos e com mobilidade reduzida. Percebe-se também que, no processo durante o trabalho continuado, cada instituição busca soluções particulares para superar as barreiras. A orientação de instituições como o Instituto Brasileiro para Segurança do Paciente (IBSP)^16^ pode aprimorar a busca de soluções, visando, por fim, o monitoramento em tempo real da prevenção de TEV e intervenções para reduzir os eventos e reinternações por TEV.
